# Improved drug targeting to liver tumor by sorafenib-loaded folate-decorated bovine serum albumin nanoparticles

**DOI:** 10.1080/10717544.2018.1561766

**Published:** 2019-02-11

**Authors:** Haipeng Wang, Shuilin Sun, Yu Zhang, Jiayi Wang, Shouhua Zhang, Xuebing Yao, Ling Chen, Zhen Gao, Baogang Xie

**Affiliations:** Department of Infectious Diseases, The Second Affiliated Hospital of Nanchang University, School of Pharmaceutical Science, Nanchang University, NanchangPR China

**Keywords:** Sorafenib, albumin nanoparticles, folate, active targeting, liver tumor

## Abstract

**Background:** To prepare sorafenib-loaded folate-decorated bovine serum nanoparticles (FA-SRF-BSANPs) and investigate their effect on the tumor targeting.

**Methods:** The nanoparticles were characterized and evaluated by in vivo and in vitro experiments.

**Results:** SRF-loaded BSA nanoparticles (SRF-BSANPs) was first prepared and modified with folic acid by chemical coupling to obtain FA-SRF-BSANPs. The average particle size, zeta potential, entrapment efficiency, and drug loading of the optimized FA-SRF-BSANPs were 158.00 nm, −16.27 mV, 77.25%, and 7.73%, respectively. The stability test showed that FA-SRF-BSANPs remained stable for more than 1 month at room temperature. The TEM analysis showed that the surface of FA-SRF-BSANPs was nearly spherical. XRD analysis showed that the drug existed in. the nanoparticles in an amorphous state. FA-SRF-BSANPs can promote the intracellular uptake of hepatoma cells (SMMC-7721) with the strongest inhibitory effect compared with SRF-BSANPs and sorafenib solution. Furthermore, the tumor targeting of FA-SRF-BSANPs (*C*_tumor_/*C*_blood_, 0.666 ± 0.053) was significantly higher than those of SRF-BSANPs (*C*_tumor_/*C*_blood_, 0.560 ± 0.083) and sorafenib-solution (*C*_tumor_/*C*_blood_, 0.410 ± 0.038) in nude mice with liver cancer.

**Conclusion:** FA-modified albumin nanoparticles are good carriers for delivering SRF to the tumor tissue, which can improve the therapeutic effect and reduce the side effects of the drug.

## Introduction

**Figure 1.**
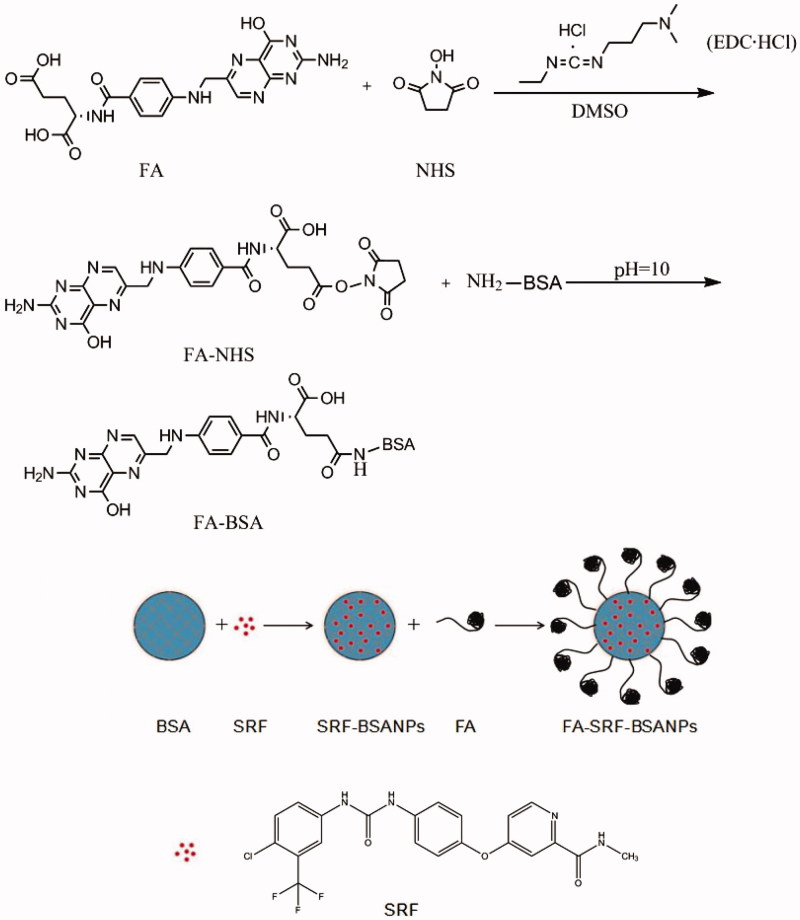
Preparation schematic of FA-SRF-BSANPs.

Sorafenib (SRF, Figure 1) is a multi-target kinase inhibitor, which is a first-line drug for the treatment of advanced liver cancer. It has obvious antitumor activity (Minami et al. 2008; Kudo 2012). Its mechanism of action is to inhibit the proliferation of tumor cells by inhibiting the receptor tyrosine kinase KIT and FLT-3 and the Raf/MEK/ERK serine/threonine kinase pathway (Strumberg et al. 2007; Miller et al. 2009; Abdel-Rahman & Elsayed 2013; Shimada et al. 2014). It inhibits neoplastic angiopoiesis by inhibiting the upstream VEGFR and PDGFR and the downstream Raf/MEK/ERK pathway. However, its low oral bioavailability (Liu et al. 2016) and side reactions (Strumberg 2012) greatly limit its clinical application. Therefore, developing a new and effective targeting drug delivery system is urgent to increase the drug concentration in the tumor and to reduce the effect of the drug on normal tissue cells, thereby improving the antitumor effect of the drug.

Folate (FA) can enter cells through a receptor-mediated endocytosis pathway. The folic acid receptor (FR) is expressed in the lungs, glands, kidney, choroid plexus, and placenta at low level in physiological state, and it is highly expressed in tumor tissue with 100–300 times higher than that in normal tissues (Weitman et al. 1992; Ross et al. 1994; Sudimack & Lee 2000). The endocytosis mediated by FR can be used to absorb the FA, FA conjugate, and FA antagonists effectively. Therefore, the FR is a good target for the target delivery system for many solid tumors (Lu & Low 2002).

As a first-line drug for treatment of advanced liver tumors, very few studies on active targeting preparations for SRF have been reported. In previous reports, Li et al. (2015) developed a nanosized SRF/FA/PEG-PLGA-NP with both anticancer and magnetic resonance properties, which can improve the antitumor activity in vitro, but this conclusion was only verified at the cellular level. Zhang et al. (2013) prepared FA-functionalized polymeric micelles loaded with SPIONs and SRF, which can increase the concentration of SRF in HepG2 cells, but no results in vivo studies were presented.

In the current study, SRF-loaded FA-decorated bovine serum nanoparticles (FA-SRF-BSANPs) were prepared through loading with bovine serum albumin (BSA) as the drug carrier and were modified with FA by chemical coupling. FA-SRF-BSANPs were characterized, and the active targeting functions of it were evaluated by in vivo and in vitro experiments, including cytotoxicity analysis, cell uptake test, liver tumor targeting evaluation, and so on.

## Materials and methods

### Materials

SRF was kindly provided by the Gz Eastbang Pharmaceutical Technology Co., Ltd. (Guangdong, China). FA, *N*-hydroxysuccinimide (NHS), and 1-ethyl-3-(3-dimethyllamino propyl) carbodiimide hydrochloride were purchased from Chengdu Aikeda Chemical Reagent Co., Ltd. (Chengdu, China). Megestrol acetate (internal standard, IS) was purchased from the TiXiAi Chemical Company (Shanghai, China). All animals in this experiment were from the Department of Animal Science, Nanchang University. 

### Preparation of SRF-BSANPs

BSA (50.0 mg) was weighted and dissolved in 10.0 mL of normal saline to form 0.5% (*w*/*v*) carrier solution. The pH of the carrier solution was adjusted to 9.0 using 0.2 mol/L NaOH solution. An appropriate amount of SRF was dissolved in ethanol (12.0 mg/mL) as the oil phase. At room temperature, 417.0 µL of the oil phase was added dropwise to carrier solution and stirring for 1.0 h at 500 rpm/min, then 0.2 mL of 0.5% glutaraldehyde was added to the system and continuously stirred for 3 h. In the end, SRF-BSANPs were obtained.

### Preparation of FA-SRF-BSANPs

The FA-SRF-BSANPs were prepared on the basis of SRF-BSANPs by the carboxyl group of FA amidatint with the active amine on the surface of albumin. The specific preparation process was as follows: (1) Preparation of folic acid active ester (FA-NHS) according to previous literature (Zhang et al. 2014) with slight modification. Briefly, 0.50 g of FA was dissolved in 15.0 mL of dimethyl sulfoxide (DMSO) containing 0.45 g of NHS and 0.25 g of EDC·HCl. The mixture was allowed for stirring for 24 h at room temperature and dark environment. Insoluble impurities were removed through filtration. The filtrate was mixed with two times volume of acetone and ether mixture (*V*:*V* = 30:70) to precipitate FA-NHS (Wang et al. 2014; Dubey et al. 2015) and filtrated to obtain the yellow precipitate. After washing three times with ether and vacuum drying, light-yellow FA-NHS was obtained. (2) The FA-NHS was dissolved in 1.0 mL of sodium carbonate/sodium bicarbonate buffer solution (pH 10.0). Newly prepared SRF-BSANPs (10.0 mL) was obtained and adjusted to pH 10.0 using 0.2 mol/L NaOH solution. FA-NHS solution was added dropwise to SRF-BSANPs, and FA-SRF-BSANPs were obtained after stirring for a period of time at room temperature and dark environment. The flowchart for the preparation of nanoparticles is shown in Supplementary Figure S1. The FA-SRF-BSANPs were placed in a dialysis bag (MWCO 3500 Da) and dialyzed for 60 h with phosphate buffered solution (PBS, pH 7.4). The dialysate was replaced every 3 h to remove excessive FA-NHS. The purified FA-SRF-BSANPs suspension was freeze dried to obtain FA-SRF-BSANPs powder.

### Physicochemical characterization of nanoparticles

#### Evaluation of FA content in FA-SRF-BSANPs

The successful coupling of FA and albumin is the key to this experiment. It was characterized by Fourier Transform infrared spectroscopy (FTIR) and proton nuclear magnetic resonance (^1^H NMR).

FTIR: FA powder, SRF-BSANPs freeze-dried powder, and FA-SRF-BSANPs freeze-dried powder was mixed with KBr powder and made into thin slices. The thin slices were then analyzed using FTIR-8400 infrared spectrophotometer (SHIMADZU, Japan).

^1^H NMR: Appropriate amount of FA, BSA, and FA-SRF-BSANPs freeze-dried powder were dissolved in deuterated DMSO (containing 0.03% TMS). After centrifugation at 13,000 rpm for 5 min, the supernatant was transferred into a 5 mm NMR tube. The samples were analyzed using NMR analyzer Brucker A-Vance-600 (Brucker, Switzerland).

FA working curve for quantification: the amount of FA was accurately weighed and dissolved in deuterium DMSO, and then the FA solution with concentrations of 1.0 2.0, 4.0, 6.0, and 8.0 mg/mL was tested for ^1^H NMR. After adjusting the baseline and phase, the peaks of TMS and FA (a multiple peak between 2 ppm and 2.1 ppm) were integrated. The ratio of the area of FA to that of TMS was recorded as A, FA concentration C was used as the *x*-axis, and A was the *y*-axis to develop the regression equation.

Three samples of FA-SRF-BSANPs freeze-dried powder prepared from the same batch were dissolved in deuterated DMSO and analyzed by ^1^H NMR. According to the above integration method and the working curve, the FA amount in FA-SRF-BSANPs was calculated.

#### Particle size, zeta potential, encapsulation efficiency, and drug loading

The particle size and zeta potential of FA-SRF-BSANPs were detected using Malvern Mastersizer (PSA NANO2590, Malvern Instruments, Malvern, UK). Entrapment efficiency and drug loading were determined by high speed centrifugation method (Dreis et al. 2007). The concentration of free SRF in the supernatant was determined by HPLC method (Wang et al. 2018).

#### Surface morphology

After dilution to a suitable concentration, nanoparticles were dropped onto copper grids and negatively stained with 2.0% phosphotungstic acid. The morphology of SRF-BSANPs and FA-SRF-BSANPs were observed under a transmission electron microscope (TEM, JEM-2100, JEOL, Tokyo, Japan).

#### X-ray diffraction (XRD) analysis

The powder XRD patterns of FA, BSA, SRF, physical mixture (FA, BSA, and SRF), and FA-SRF-BSANPs were investigated using X-ray diffractometer. (Diffraction angle 5°–50°, scanning speed 5°/min, CuKa diffraction source; D8 ADVANCE, Germany BRUKER).

#### *In vitro* cytotoxicity assay

Human normal hepatocyte LO2 cell line and liver cancer cell line SMMC-7721 were purchased from Beijing Solarbio Science & Technology Co., Ltd. and they were incubated in DMEM medium (including 10% fetal bovine serum, 100 U/mL penicillin, 100 U/mL streptomycin) in cell culture incubator (37 °C, 5% CO_2_).

LO2 and SMMC-7721 cells were inoculated with the density of (5 × 10^3^/well) in 96 well plates and incubated for 24 h. The cell culture media without drug were used as the control group, and the SRF solution, SRF-BSANPs, and FA-SRF-BSANPs were used as the experimental group. After the cells were adhered, the old medium was removed, and 0.2 mL of medium containing drug was added to each well (three SRF preparations were diluted to 60.0, 40.0, and 20.0 µg/mL with the medium, respectively.) and incubated for 24 h. Then 15.0 µL MTT solution (5 mg/mL) was added to each well in the dark. The medium was removed after 4 h, and the DMSO was added to dissolve formazan, followed by measurement of the absorbance at 490 nm (A) with DNM-9602A microplate reader (Beijing PERLONG medical company) to calculate the inhibition ratio.

$$\text{Cell}\text{inhibition}\text{ratio}\left(\%\right)=1-A_{\text{experimental}\text{group}}/A_{\text{control}\text{group}}$$

#### Hepatoma carcinoma cell uptake experiment

In order to investigate the uptake of albumin nanoparticles and FA modified albumin nanoparticles in hepatoma cells, fluorescein isothiocyanate (FITC), instead of SRF, was used to prepare FITC-BSANPs and FA-FITC-BSANPs. The preparation process of FITC-BSANPs and FA-FITC-BSANPs were the same as that of SRF-BSANPs and FA-SRF-BSANPs.

SMMC-7721 cells from the logarithmic growth period were digested with trypsin and was inoculated in the six-well plate (1 × 10^5^/well) and cultured in the incubator. After the cells were adhered, the old medium was removed, and 3.0 mL of medium containing nanoparticles (FITC-BSANPs and FA-FITC-BSANPs 20.0 µg/mL) were added to the wells and incubated for 2 h. The cells were washed three times with PBS and were observed and photographed under the fluorescent inverted microscope (OLYMPUS IX71 inverted fluorescence microscope, Beijing OLYMPUS Sales Service Co., Ltd.).

In addition, another 3.0 mL of medium containing nanoparticles (FITC-BSANPs and FA-FITC-BSANPs were diluted to 20.0, 10.0, and 5 μg/mL with the medium) were added to the wells and incubated for 2 h. The cells were washed three times with PBS and digested with trypsin. After centrifugation at 1000 rpm for 5.0 min, the obtained cell precipitation was lysed in 4.0 mL of methanol with ultrasound, and centrifuged again. The fluorescence intensity in the supernatant was measured using fluorescence spectrophotometer (*λ*_ex_=494.0 nm, *λ*_em_=518.0 nm, PerkinElmer LS55 fluorescence spectrophotometer, Perkin Elmer Enterprise Management Co., Ltd.)

#### Liver targeting of FA-SRF-BSANPs in healthy rats

Animal experimentation followed the approval of the Animal Care Committee of Nanchang University in this study. Forty-five healthy adult female SD rats were randomly divided into three groups. Each rat was given orally a single dose (7.5 mg/kg) of SRF suspension, SRF-BSANPs, or FA-SRF-BSANPs. Three rats were sacrificed in each group at 2.0, 6.0, 10.0 24.0, and 58.0 h, the blood and liver tissues were collected. The serum and liver tissues were stored at −40 °C.

Drug targeting index (DTI) and drug selectivity index (DSI) were used as indicators to quantitatively evaluate the distribution characteristics of target preparations in vivo. DTI was used to compare the differences in the tendency of different preparations to an organ. DSI was used to compare the distribution of target preparations between target organs and nontarget organs at a certain time. The formulas for both of them were as follows:

$$\text{DTI}=\frac{\text{Drug}\text{content}\text{of}\text{the}\text{liver}\text{tissue}\text{at}T\text{moment}\text{after}\text{the}\text{administration}\text{of}\text{target}\text{preparation}}{\text{Drug}\text{content}\text{of}\text{liver}\text{tissue}\text{at}T\text{moment}\text{after}\text{administration}\text{of}\text{nontargeted}\text{preparations}}$$

$$\text{DSI}=\frac{\text{Drug}\text{content}\text{of}\text{liver}\text{tissue}\text{at}T\text{moment}}{\text{Drug}\text{content}\text{of}\text{blood}\text{at}T\text{moment}}$$

#### Tumor targeting of FA-SRF-BSANPs in nude mice

Four-week-old male athymic nude mice were obtained from the Department of Animal Science of Nanchang University and adapted to the environment for a week. A total of 0.1 mL (1 × 10^7^) of SMMC-7721 cells of logarithmic growth phase was subcutaneously injected into the right hind limb of each nude mice, and the size of the tumor was observed. When the tumor grew to approximately 1 cm^3^, the nude mice were randomly divided into three groups, five rats in each group, with a single intraperitoneal injection of 7.5 mg/kg SRF-suspension, SRF-BSANPs, or FA-SRF-BSANPs. At 1.0 h after administration, the nude mice were killed, and blood, liver, as well as tumor tissues were collected. A small amount of tumor tissue was fixed with 4% paraformaldehyde for HE staining. Serum, liver, and tumor tissue were kept at −40 °C for further SRF quantification. The methods for biological sample treatment and quantification of SRF concentration were referred to our previous study (Wang et al. 2018).

## Results and discussion

### Preparation of nanoparticles

In this study, albumin nanoparticles were prepared by an improved self-assembly method. This method is simple and convenient without special equipment requirements. In order to obtain high-quality nanoparticles, five factors, including the concentration of the carrier solution, pH value, amount of the oil phase solution, amount of glutaraldehyde, and curing time of the crosslinking, were investigated in this experiment. The optimum conditions were described. In addition, in order to obtain the nanoparticles with high FA coupling amount during the preparation of FA-SRF-BSANPs, we also optimized the amount of FA-NHS and the reaction time. The results showed that FA coupling was the highest when the dosage of FA-NHS was 5.0 mg, and the reaction time was 8 h.

### Evaluation of FA content in FA-SRF-BSANPs

The infrared spectra of FA, SRF-BSANPs, and FA-SRF-BSANPs are shown in Supplementary Figure S2(a). In the infrared spectrum of FA-SRF-BSANPs, the characteristic peaks of FA belonging to (–OH) disappeared at 932 cm^−1^, and the amino characteristic peak of BSA at 612 cm^−1^ on the lysine was not obvious, and probably the reaction occurred between the amino group and the carboxyl group. In addition, three characteristic peaks of 1664 cm^−1^ (C = O), 1548 cm^−1^ (N–H), and 1036 cm^−1^ (C–N) represented the new amide bonds formed by the coupling of FA and BSA in the infrared spectrum of FA-SRF-BSANPs. According to the above data, FA was successfully coupled with BSA.

The ^1^H NMR diagram of FA, SRF-BSANPs, and FA-SRF-BSANPs is shown in Supplementary Figure S2(b). We can see that FA and FA-SRF-BSANPs had multiple peaks at 1.85–1.95 2.00–2.10, and 2.25–2.35 ppm, while SRF-BSANPs had no multiple peaks at these three sites, indicating that FA and BSA were successfully coupled. In addition, the characteristic peak (11.40 ppm) of active H on FA disappeared in the ^1^H NMR pattern of FA-SRF-BSANPs, which also demonstrated that FA reacted with BSA. According to the standard curve equation of FA (Supplementary Figure S3, A = 0.5317C + 0.149, *R*^2^=0.9989.), the FA coupling capacity was calculated to be 62.46 ± 1.98 µg (FA)/mg (BSA).

### Particle size, zeta potential, entrapment efficiency, drug loading, and stability of the nanoparticles

The morphological structures of SRF-BSANPs and FA-SRF-BSANPs were observed under a transmission electron microscope (Supplementary Figure S4(b,d)). According to the diagram, the nanoparticles were spherical and smooth in surface.

The particle size, zeta potential, entrapment efficiency, and drug loading of SRF-BSANPs and FA-SRF-BSANPs are shown in Table 1. The average particle size of SRF-BSANPs was 129.42 ± 0.61 nm, and the size of albumin nanoparticles increased slightly (158.00 ± 2.43 nm) after the coupling of FA, which was consistent with the literature (Dubey et al. 2015). The particle size distributions of SRF-BSANPs and FA-SRF-BSANPs are shown in Supplementary Figure S4(a,c), which had relatively uniform particle size distribution.

**Table 1.** Average particle size, zeta potential, entrapment efficiency, and drug loading of FA-SRF-BSANPs and SRF-BSANPs. (*n* = 3, mean ± SD).

Formulations	Particle size (nm)	Zeta potential (mV)	EE%	DL%
SRF-BSANPs	129.42 ± 0.61	−16.48 ± 1.58	85.13 ± 1.56	8.51 ± 0.16
FA-SRF-BSANPs	158.00 ± 2.43	−16.27 ± 0.97	77.25 ± 0.97	7.72 ± 0.10

The physical stability of the prepared FA-SRF-BSANPs was evaluated in brown bottle over 2 months at 25 ± 2 °C. FA-SRF-BSANPs were sampled on the 1st, 15th, 30th, 45th, and 60th day of storage. The stability of nanaparticles was evaluated by the appearance, EE, and particle size. The results are shown in Supplementary Table T1. During 30 days of storage, the appearance, EE, and particle size of FA-SRF-BSANPs did not change substantially, indicating that the FA-SRF-BSANPs are stable at room temperature for 1 month. 

### XRD analysis

The results of XRD analyses on FA, BSA, SRF, physical mixture, and FA-SRF-BSANPs are shown in Supplementary Figure S5. The SRF had sharp characteristic peaks at 2*θ* = 13.3°, 2*θ* = 17.7°, and 2*θ* = 22.9°, indicating that SRF exhibited a crystal structure, and the three characteristic peaks can be observed in the XRD Atlas of physical mixture. However, the three characteristic peaks in the XRD Atlas of freeze-dried FA-SRF-BSANPs powder completely disappeared, which indicated that SRF was amorphously enclosed in the nanoparticles. 

### *In vitro* cytotoxicity assay of nanoparticles

**Figure 2.**
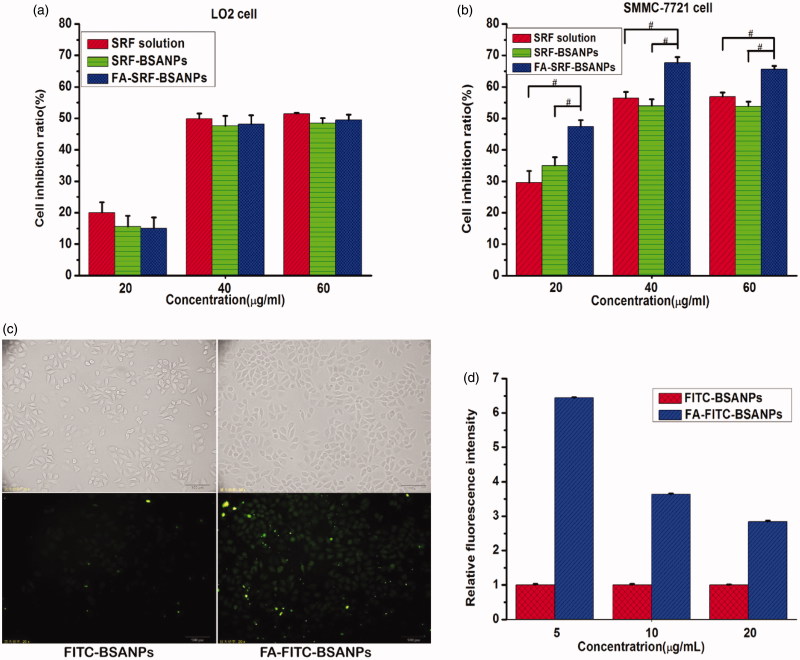
Cell inhibition ratio on three concentration levels of SRF solution, SRF- BSANPs, and FA-SRF-BSANPs against (a) LO2 cell lines or (b) SMMC-7721 cell lines after incubation for 24 h; (c) Cellular uptake of FITC-BSANPs and FA-FITC-BSANPs by SMMC-7721 cells. (d) Histogram of relative quantitative analysis of SMMC-7721 cell uptake of FITC-BSANPs and FA-FITC-BSANPs. (Mean ± SE^#^ indicates a statistically significant difference between two groups *p* < .05, independent sample *t*-test).

The results of cytotoxicity test are shown in Figure 2(a,b). As shown in Figure 2(a), the toxicity of SRF-solution on LO2 cells was slightly stronger than that of SRF-BSANPs and FA-SRF-BSANPs under the same concentrations, but no statistical difference was observed. Interestingly, when SRF concentration was at 40.0 µg/mL, the inhibition rates of SRF-solution, SRF-BSANPs, and FA-SRF-BSANPs to LO2 cells (49.93%, 47.59%, and 48.18%, respectively) were significantly stronger than that in 20.0 µg/mL (the inhibition rates: 19.96%, 15.63%, 15.01%, respectively), but when SRF concentration was increased to 60 µg/mL, the cell inhibition rate (51.42%, 48.47%, and 49.47%, respectively) was not significantly increased. This might be because the optimal concentration was between 40.0 and 60.0 µg/mL.

Figure 2(b) shows that FA-SRF-BSANPs exerted the highest SMMC-7721 cell inhibition rate at three concentration levels, compared with SRF-solution and SRF-BSANPs. The FA-modified SRF-BSANPs had significant targeting ability to hepatoma cells, which can enhance the anti-cancer effect of SRF in vivo. 

### Uptake of nanoparticles in hepatoma carcinoma cell

Figure 2(c,d) shows that the fluorescence intensity of FA-FITC-BSANPs group was obviously stronger than that of FITC-BSANPs group. The fluorescence intensity of the FA-FITC-BSANP group was 2.84, 3.63, and 6.43 times that of the FITC-BSANP group at concentrations of 20.0, 10.0, and 5.0 µg/mL, respectively. The uptake of FA-FITC-BSANPs by SMMC-7721 cells was greater than that of FITC-BSANPs, further demonstrating that FA modified albumin nanoparticles had good targeting to hepatoma cells.

### Investigation of liver targeting of FA-SRF-BSANPs in healthy rats

The values of DTI and DSI after single oral administration of SRF-BSANPs, FA-SRF-BSANPs, and SRF-suspension are shown in Table 2. The mean values of DTI in the SRF-BSANPs group and FA-SRF-BSANPs group were 26.85 ± 7.62 and 24.21 ± 7.94, respectively, which showed that the two nanoparticles exhibited good liver targeting compared with SRF-suspension. Table 2 also shows that both SRF-BSANPs and FA-SRF-BSANPs had higher DSI values at all time points after oral administration than those in SRF-suspension group. The average values of DSI in SRF-BSANPs group (6.14 ± 0.69) and FA-SRF-BSANPs group (6.93 ± 0.43) were 2.79 and 3.15 times those of SRF-suspension group (2.20 ± 0.48), respectively. SRF-BSANPs and FA-SRF-BSANPs exhibited good targeting in rat liver compared with SRF-suspension, but no difference was observed between SRF-BSANPs and FA-SRF-BSANPs.

**Table 2.** DTI and DSI values after oral administration of SRF-BSANPs, FA-SRF-BSANPs and SRF-Suspensions in healthy rats (7.5 mg/kg, *n* = 3).

Time (hour)		2	6	10	24	58	Mean ± SE
DTI	SRF-BSANPs	41.77	47.85	11.18	21.64	11.81	26.85 ± 7.62
	FA-SRF-BSANPs	31.93	51.11	8.80	9.34	19.89	24.21 ± 7.94
DSI	SRF-Suspension	2.40	3.97	1.39	1.50	1.73	2.20 ± 0.48
	SRF-BSANPs	7.94	6.98	4.90	6.67	4.23	6.14 ± 0.69
	FA-SRF-BSANPs	6.66	6.91	8.56	6.03	6.47	6.93 ± 0.43

### Investigation of tumor targeting of FA-SRF-BSANPs in nude mice

**Figure 3.**
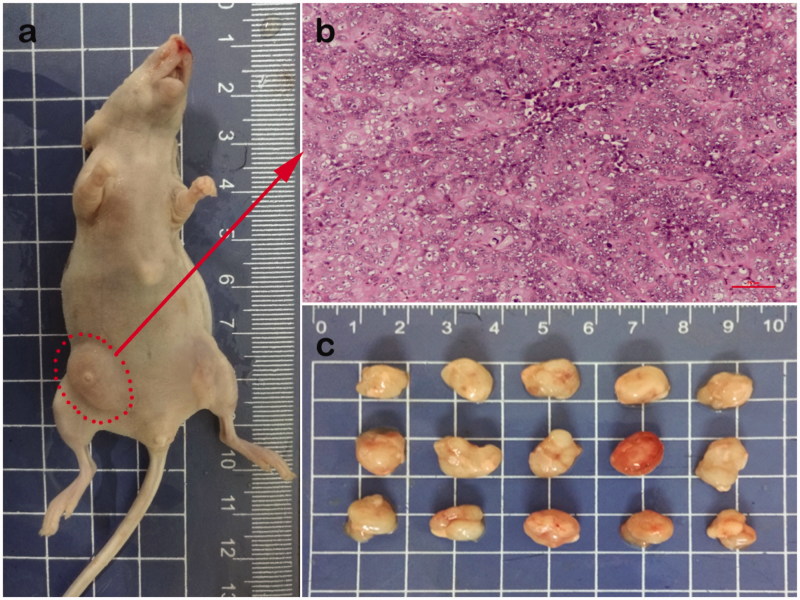
(a) Image of nude mice with liver cancer (b) Tumor tissue sections (stained by H&E) (c) Tumor tissues.

**Figure 4.**
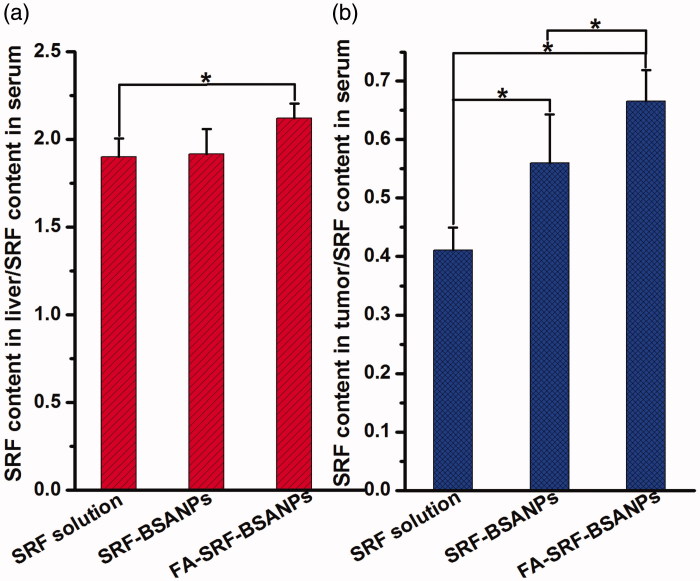
Ratio of SRF content in liver (a) or tumor tissue (b) and SRF content in serum after IP administration of SRF-Solution, SRF-BSANPs, or FA-SRF-BSANPs (Mean ± SD, *n* = 5). *Indicates a statistically significant difference between two groups. (*p* < .05, independent sample *t*-test).

In the current study, the nude mice model of liver tumor was successfully established (Figure 3). The nude mice were randomly divided into three groups. The mice were given SRF-solution, SRF-BSANPs, and FA-SRF-BSANPs in a single IP administration. After 1 h, the nude mice were killed and samples of blood, liver, and tumor were collected. The content of SRF in each sample was measured. In order to more intuitively characterize the targeting of SRF preparations, the ratio of the SRF concentration in the nude mouse liver or tumor sample to the SRF concentration in the blood sample (*C*_liver_/*C*_blood_ or *C*_tumor_/*C*_blood_) was analyzed, and the results are shown in Figure 4. The *C*_liver_/*C*_blood_ of FA-SRF-BSANPs group or SRF-BSANPs group was significantly greater than that of the SRF-solution group, but no statistical difference was observed between the FA-SRF-BSANPs group and the SRF-BSANPs group, indicating that FA-SRF-BSANPs or SRF-BSANPs had a certain liver tissue targeting. However, the FA did not increase the drug concentration in normal liver tissue, which was consistent with the results of the nontumor-bearing healthy rats. More importantly, the *C*_tumor_/*C*_blood_ values of SRF-BSANPs group (0.560 ± 0.083) and FA-SRF-BSANPs group (0.666 ± 0.053) were significantly higher than those of the SRF-solution group (0.410 ± 0.038). The difference between the SRF-BSANPs group and the FA-SRF-BSANPs group was significant (*p* < .05), indicating that FA-SRF-BSANPs and SRF-BSANPs had obvious targeting of tumor tissue, and FA-SRF-BSANPs had a stronger targeting effect on tumor tissue, and can significantly increase the concentration of SRF in tumor tissue.

Self-assembled polymer systems at the nanometer scale have attracted much attention especially in biomedical fields (Zhao et al. 2014; Li et al. 2016). Drugs are dispersed, encapsulated, and adsorbed on polymer particles. They are released through cystic wall leaching, permeation, and diffusion and can be released by the dissolution of the matrix itself. It has the advantages of targeting, releasing, increasing the absorption and bioavailability of drugs, increasing solubility of insoluble drugs, improving drug stability, and reducing adverse reactions (Wang et al. 2015). Since FDA approved the clinical usage of the albumin-bound paclitaxel nanoparticle suspension injection in 2005, albumin nanoparticles have received considerable clinical attention for targeting drug carriers (Zhao et al. 2015). Albumin is an endogenous substance and has the advantages of biodegradation, nontoxicity, harmlessness, non-immunogenicity, easy to purify, and solubility in water. Drugs can be physically adsorbed or covalently bound to the surface of albumin nanoparticles (Hawkins et al. 2008; Kim et al. 2011; Altintas et al. 2013). It has been recognized as an ideal carrier material for preparing nanoparticles. In addition, the active groups on the surface of the albumin nanoparticles are the effective parts of the chemical modification, and various target ligands can be coupled with them (Steinhauser et al. 2008; Ulbrich et al. 2009; Kouchakzadeh et al. 2012; Kouchakzadeh et al. 2013), thereby making the nanoparticles multifunctional.

FA coupling and FA antagonists may be effectively used to the target delivery system in many tumor cells (Sudimack & Lee 2000; Pan & Lee 2004). Aim to enhance the antitumor effect of SRF, we prepared the FA-SRF-BSANPs by amidation of FA and albumin nanoparticles under alkaline conditions, giving the nanoparticles an active targeting property into the tumor cells. The successful coupling of FA and BSA was proved by FTIR and ^1^H NMR techniques. Our results showed that the coupling amount of FA to BSA was the highest when the ratio of FA-NHS to BSA was 5:50 and the reaction time was 8 h, the FA coupling capacity was 62.46 ± 1.98 µg (FA)/mg (BSA).

A series of optimization and characterization of the nanoparticles were carried out. An approximate spherical nanoparticle with a particle size of 158.00 ± 2.43 nm and a zeta potential of −16.27 ± 0.97 mV was obtained. The particles with zeta potential values ranging from −30 mV to +30 mV never settle down quickly and should be stored in powder form or suspended in saline solution or water and shaken well before administration (Basu et al. 2012). Under transmission electron microscope, FA-SRF-BSANPs was observed to be spherical and uniform in size. XRD results showed that SRF was encapsulated in nanoparticles in amorphous form. The encapsulation efficiency and drug loading of the nanoparticles were high, and their physical stability could be stored for more than one month at room temperature. To the best of our knowledge, this is the first report on active-targeting nanoparticles with improved drug targeting to the liver tumor.

LO2 cells were normal human hepatocytes, and the FA receptors were expressed at low levels on its surface. Therefore, no significant difference was observed in the inhibitory effect of SRF-BSANPs and FA-SRF-BSANPs on LO2 cells. SMMC-7721 cells were human hepatoma cells, and FA receptors were highly expressed on their surface. The FA molecules on the surface of FA-SRF-BSANPs specifically bound to the FA receptor on the surface of SMMC-7721 cells, thereby delivering drugs to HCC cells. In addition, to understand the difference of cellular uptake of nanoparticles in hepatoma carcinoma cell, the uptake of FITC-BSANPs and FA-FITC-BSANPs by SMMC-7721 was studied in this experiment. FA-FITC-BSANPs showed much higher fluorescence intensity in SMMC-7721 cells in comparison to FITC-BSANPs. Therefore, compared with SRF-BSANPs, higher cellular uptake and the inhibitory effect of FA-SRF-BSANPs on HCC cells were observed, demonstrating that FA modified albumin nanoparticles had good targeting to hepatoma cells.

The liver and tumor targeting of SRF-BSANPs and FA-SRF-BSANPs was studied in this study. DSI and DTI values were chosen as indicators to quantitatively evaluate the distribution characteristics of target preparations in vivo. When the DSI value is more than 1.0, it indicates that the content of drugs in liver tissue is higher than that in blood at a certain time. The greater the DSI value, the better the selectivity of the preparation to liver tissue. The results showed that the DSI values of the two nanoparticles were higher than those of the suspension group at all time points after oral administration, indicating that nanoparticles exhibited good liver targeting compared with SRF-suspension. Compared with SRF-suspension, SRF-BSANPs and FA-SRF-BSANPs exhibited good liver targeting in healthy rats, but no differences between them were observed. Furthermore, the liver tumor model was successfully established by subcutaneous injection of SMMC-7721 cells in nude mice. Our results showed that the *C*_tumor_/*C*_blood_ values of the FA-SRF-BSANPs group were significantly higher than those of the SRF-solution and SRF-BSANPs group, indicating improved tumor targeting of FA-SRF-BSANPs. This is in agreement with the results of cell experiments.

## Conclusion

In this study, an FA-modified SRF albumin nanoparticle with active targeting function was successfully prepared. A series of optimization and characterization of the nanoparticles were carried out. An approximate spherical nanoparticle with a particle size of 158.00 ± 2.43 nm and a zeta potential of −16.27 ± 0.97 mV was obtained. The encapsulation efficiency and drug loading of the nanoparticles were high, and their physical stability was good. Compared with SRF-solution and SRF-BSANPs, FA-SRF-BSANPs can significantly increase the inhibitory effect of SRF on hepatoma carcinoma cells. Cellular uptake experiments also showed that FA-SRF-BSANPs significantly increased the uptake of SRF by hepatoma carcinoma cells. The tumor targeting of FA-SRF-BSANPs was significantly higher than those of SRF-BSANPs and SRF-solution in nude mice. FA-modified albumin nanoparticles were a good carrier for delivering SRF, which was relatively enriched in tumor tissue and can improve the therapeutic effect and reduce the side effects of the drug.

## Supplementary Material

WHP-20181213Supplementary_Figures_andTables.doc
