# Supplementary material for: Improved drug targeting to liver tumor by sorafenib-loaded folate-decorated bovine serum albumin nanoparticles
Source: Drug Deliv. 2019 Feb 11;26(1):89–97. doi: 10.1080/10717544.2018.1561766 (PMC6374969; doi:10.1080/10717544.2018.1561766)
Supplement: WHP-20181213Supplementary_Figures_andTables.doc [file IDRD_A_1561766_SM5176.doc]

**Supplementary Figure:**


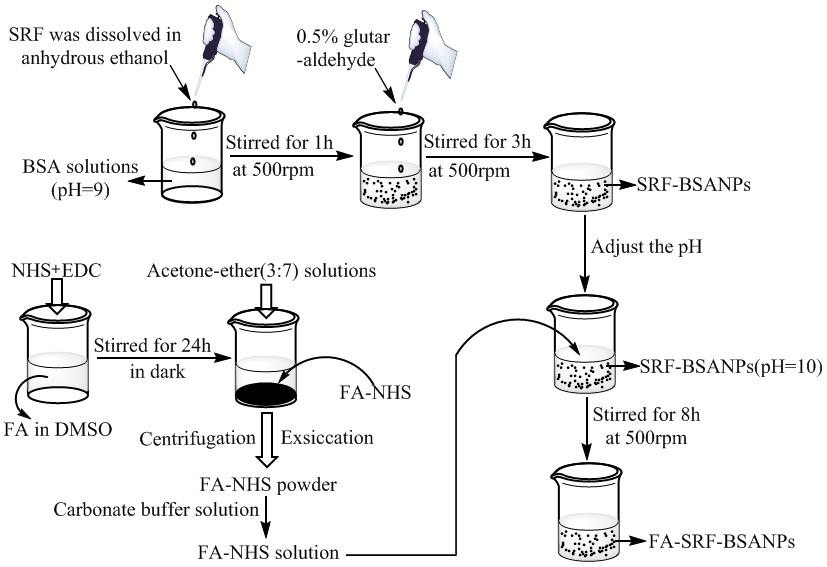


**Supplementary Figure S1.** Preparation process of FA-SRF-BSANPs.


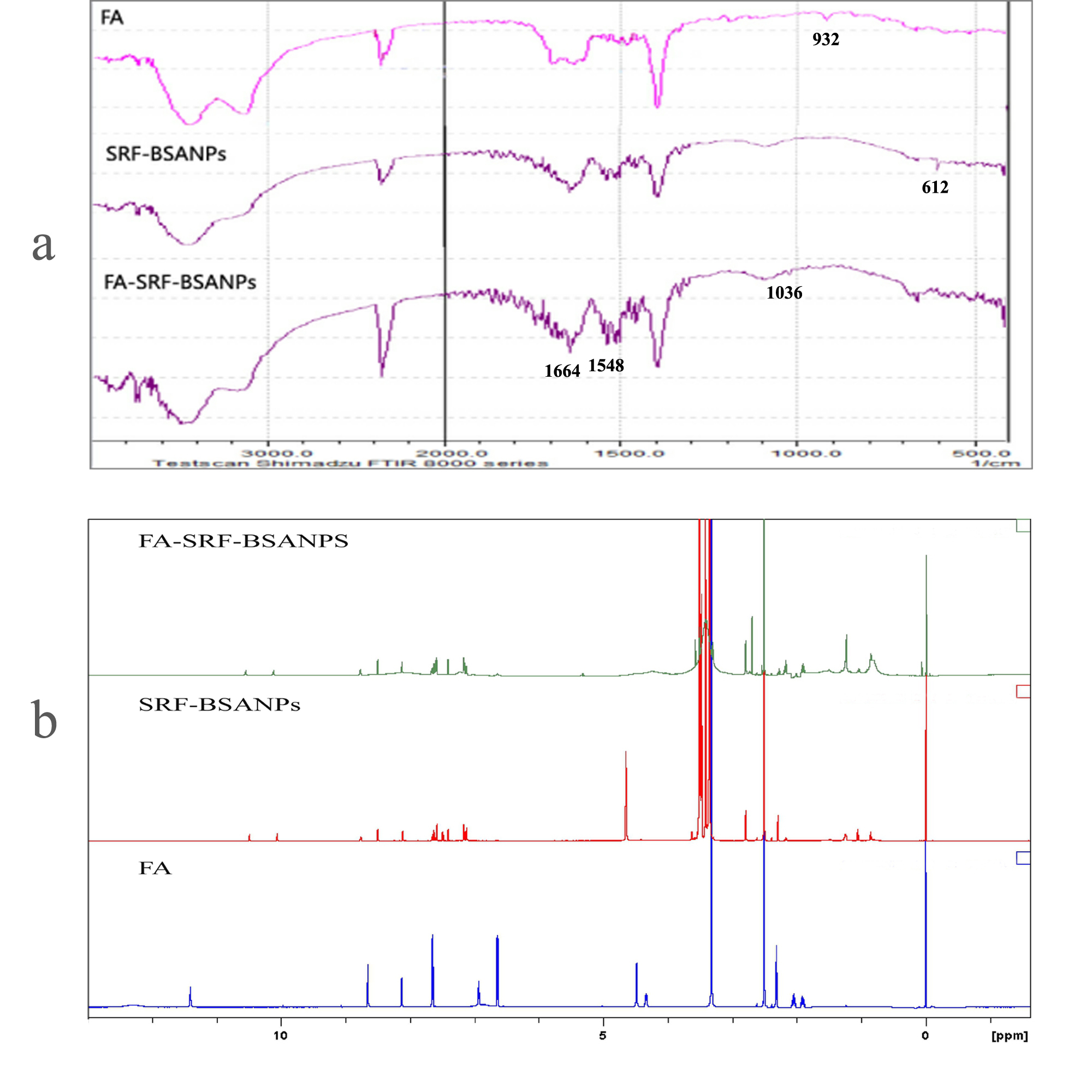


**Supplementary Figure S2. (a)** FTIR photography of FA, SRF-BSANPs, and FA-SRF-BSANPs **(b)** The ^1^H NMR photography of FA, SRF-BSANPs, and FA-SRF-BSANPs

**Supplementary Figure S3.** The calibration curve of FA.


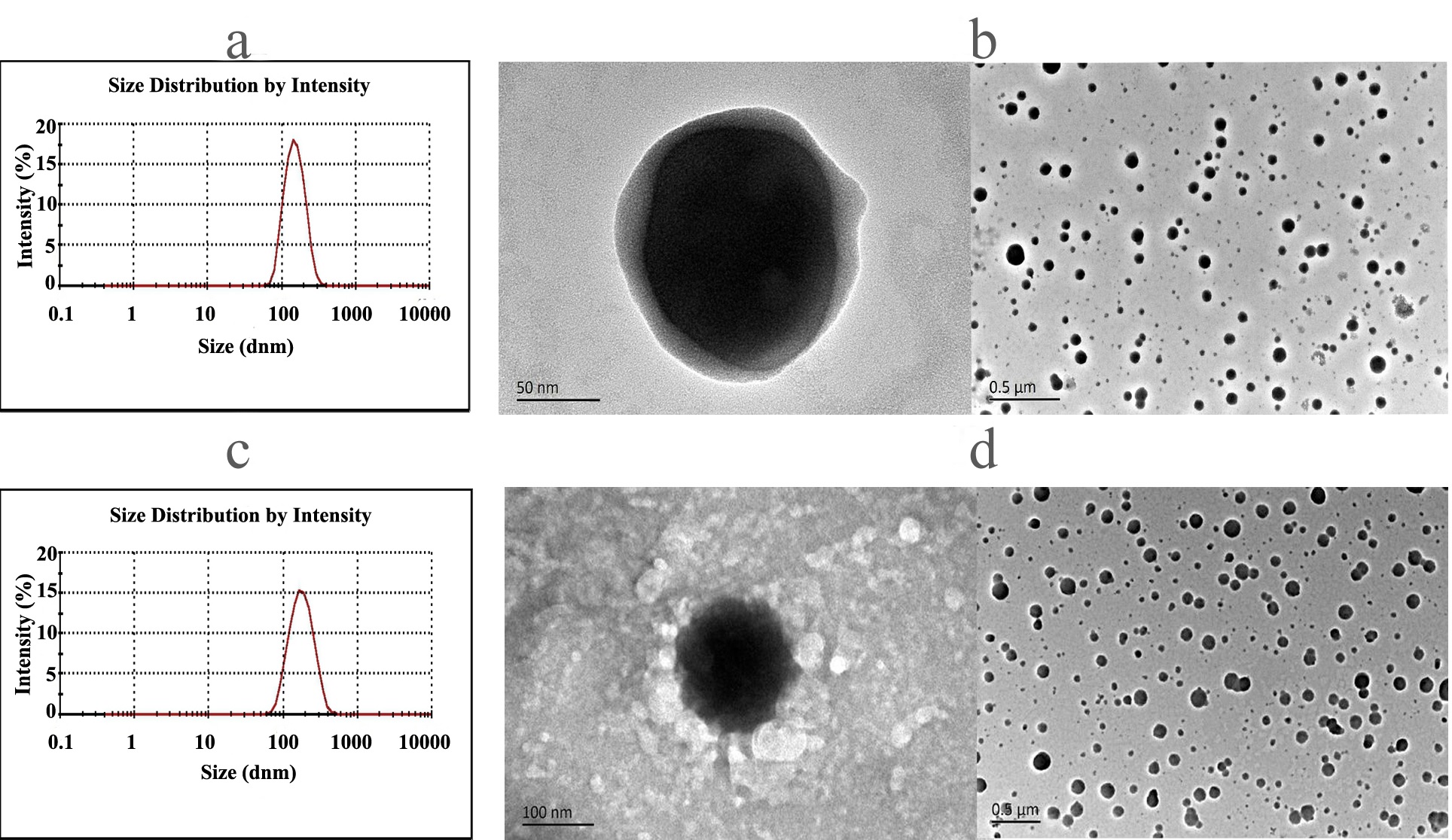


**Supplementary Figure S4.** **(a)** Particle size distribution of SRF-BSANPs; **(b)** TEM image of SRF-BSANPs; **(c)** Particle size distribution of FA-SRF-BSANPs; **(d)** TEM image of FA-SRF-BSANPs

**
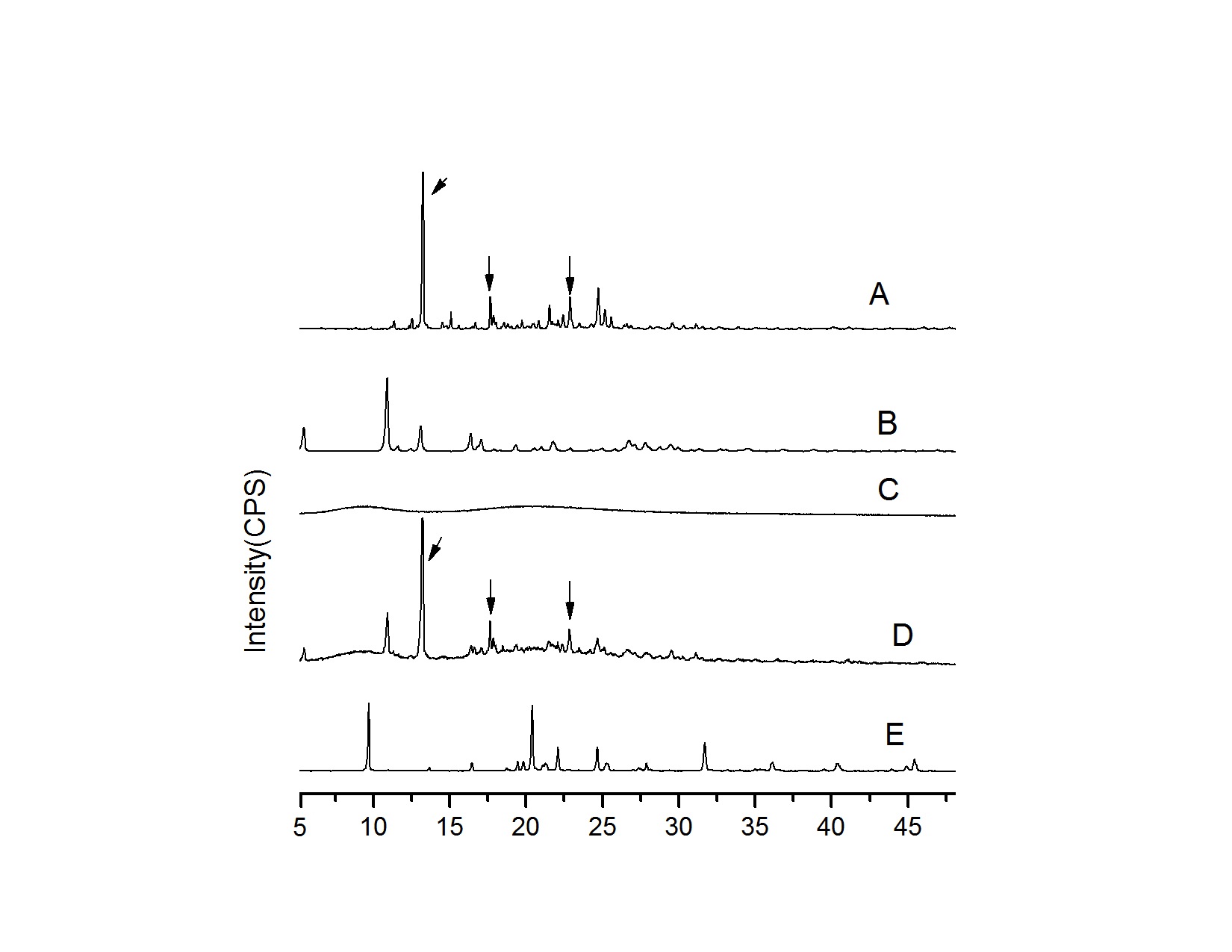
**

**Supplementary Figure S5.** XRD spectra. **(A)** SRF, **(B)** FA, **(C)** BSA, **(D)** physical mixture of SRF, FA, and BSA, **(E)** FA-SRF-BSANPs powders

**Supplementary Table T1.** The stability study of FA-SRF-BSANPs (n=3)

| Time(day) | Appearance | EE (%) | Particle size |
| --- | --- | --- | --- |
| 0 | No precipitation | 77.25±0.97 | 158.00±2.43 |
| 15 | No precipitation | 76.40±1.06 | 159.31±1.50 |
| 30 | No precipitation | 73.63±1.64 | 162.27±2.11 |
| 45 | Small amount of precipitation | 65.38±2.31 | 165.05±2.26 |
| 60 | Precipitation | 58.72±3.54 | 166.76±1.84 |
